# Anti-Inflammatory Diets in Metabolic Syndrome and Obesity: Multi-Omics Perspectives on the Interplay Between Gut Microbiota, DNA Methylation, and Adipokine Regulation—A Narrative Review

**DOI:** 10.3390/ijms27062734

**Published:** 2026-03-17

**Authors:** Karol Makiel

**Affiliations:** Department of Anatomy, Faculty of Physical Rehabilitation, University of Physical Culture, 31-571 Cracow, Poland; karol.makiel@awf.krakow.pl

**Keywords:** metabolic syndrome, gastrointestinal microbiome, DNA methylation, adipokines, diet therapy, inflammatory markers

## Abstract

An anti-inflammatory dietary pattern represents a key component of non-pharmacological management in obesity and metabolic syndrome (MetS), as it targets chronic low-grade inflammation, adipose tissue dysfunction, insulin resistance, and disturbances of the gut–metabolic axis. In the present work, we outline a framework for an “omics-based” approach that integrates data on gut microbiota composition and function (metagenomics), adipokine profiles, nutrigenomics, epigenetics, and related transcriptomic and metabolomic layers in order to enable more precise characterization of the metabolic phenotype and to support precision nutrition strategies. The proposed dietary model emphasizes the quality rather than merely the quantity of macronutrients, with particular focus on lipid profile optimization. Specifically, total fat intake is recommended to remain below 30% of total energy through the reduction in saturated fatty acids (SFA), trans fats, and excessive omega-6 fatty acids, alongside increased consumption of omega-3 PUFA (EPA/DHA) and plant-based sources of α-linolenic acid (ALA). Concurrently, greater intake of lean protein sources and low-glycemic-index carbohydrates rich in dietary fibre—particularly fermentable fractions—is recommended. The model also highlights the importance of polyphenols with antioxidant and immunomodulatory properties. To enhance feasibility and long-term adherence, recommendations are structured as flexible food substitutions rather than rigid prescriptions. Further well-designed interventional studies are required to confirm the impact of a multi-omics-based anti-inflammatory diet on both molecular and clinical endpoints.

## 1. Introduction

Metabolic syndrome (MetS) is a complex pathophysiological condition that develops primarily as a result of long-term imbalance between excessive dietary energy intake and insufficient energy expenditure. It comprises a cluster of co-occurring metabolic abnormalities, including dyslipidaemia, arterial hypertension, and insulin resistance (IR), largely driven by excessive accumulation of visceral adipose tissue. The coexistence of these disturbances contributes to numerous adverse health outcomes [1]. MetS is associated with a fivefold increased risk of developing type 2 diabetes mellitus (T2DM), a twofold higher risk of cardiovascular disease (CVD) within 5–10 years, a two- to fourfold increased risk of stroke, a three- to fourfold higher risk of myocardial infarction, and a twofold increase in mortality from these causes compared with individuals without the syndrome [2]. In developed countries, MetS affects approximately 28.4–51% of the adult population [3,4]. Both genetic predisposition and environmental factors, especially diet and low levels of physical activity, mainly contribute to the development of MetS. Lifestyle modification, especially implementation of a well-balanced diet and regular physical activity, remains the basic elements of therapeutic strategies for individuals with MetS and obesity [5,6].

Importantly, such interventions influence not only key metabolic parameters but also induce alterations in the gut microbiome, which plays a significant role in the pathogenesis of metabolic disturbances associated with MetS [7]. Despite differences in the underlying mechanisms of metabolic diseases, they are consistently linked to both shared and disorder-specific alterations in gut microbiota composition and function [8,9,10,11].

In parallel with microbiome alterations, obesity and MetS are associated with significant epigenetic modifications, including changes in DNA methylation patterns in adipose tissue and other organs involved in the regulation of energy homeostasis [12]. Patients with MetS show both global DNA methylation changes and differential methylation within promoter regions of genes critical for adipogenesis, lipid metabolism, and insulin sensitivity, such as PPARA, PPARG, ADIPOQ, and LEP. The alterations have been linked to increased insulin resistance, dyslipidaemia, and elevated risk of T2DM and CVD development [13,14]. Because epigenetic modifications are partially reversible, lifestyle-based interventions, including anti-inflammatory dietary patterns, may modulate DNA methylation and thereby influence the clinical course of metabolic disorders [14].

Obesity is considered one of the primary drivers of MetS. Chronic low-grade inflammation associated with obesity represents a key mechanism underlying metabolic homeostasis disruption induced by excessive nutrient intake. This process involves regulation of gene expression encoding cytokines, chemokines, and other inflammatory mediators through transcription factors such as nuclear factor κB (NF-κB), activator protein-1 (AP-1), nuclear factor of activated T cells (NFAT), and signal transducer and activator of transcription 3 (STAT3). These factors activate the inflammasome, a macromolecular sensor of the innate immune system, leading to activation of the caspase-1 pathway and subsequent proteolytic maturation of inflammatory mediators. Consequently, an increased secretion of pro-inflammatory cytokines takes place, including TNF-α, IL-6, CRP, and IL-1β, by M1 macrophages within white adipose tissue, accompanied by a marked reduction in anti-inflammatory mediators such as IL-10, IL-Ra, and adiponectin. The chronic inflammatory state observed in obesity disrupts not only adipose tissue secretory function but also affects the immune system, liver, brain, skeletal muscle, pancreas, and intestinal barrier [15,16].

Within this context, an anti-inflammatory dietary pattern may be viewed not only as a supportive clinical strategy, but also as a potential molecular modulator acting across interconnected biological layers relevant to MetS. By influencing nutrient quality, fatty acid composition, fibre intake, and exposure to bioactive compounds such as polyphenols, dietary patterns may affect gut microbiota composition and microbial metabolite production, intestinal barrier function, inflammatory signalling, DNA methylation processes, and adipokine secretion [14,17,18]. Accordingly, the aim of this narrative review is to discuss anti-inflammatory diets in obesity and metabolic syndrome from a multi-omics perspective, with particular emphasis on the interplay between gut microbiota, epigenetic regulation, and adipokine signalling. The following sections therefore first outline the inflammatory background of MetS and obesity, then discuss the characteristics of anti-inflammatory dietary models, and finally examine selected mechanistic links between diet, the microbiome, DNA methylation, and adipokine regulation within an integrated framework. For conceptual clarity, the multi-omics framework discussed in this review can be summarized as follows: diet constitutes the exposure layer; gut microbiota composition and microbiota-derived metabolites represent the metagenomic/metabolomic interface; inflammatory signaling and adipokine secretion reflect major functional host responses; and DNA methylation represents one of the epigenetic mechanisms through which these signals may be biologically embedded and translated into metabolic phenotype.

## 2. Chronic Inflammation in Metabolic Syndrome and Obesity

Inflammation is a fundamental biological process essential for human survival. It represents a normal and necessary physiological response to internal injury and a wide range of external stimuli, including foreign substances and tissue damage. When properly regulated, the inflammatory response facilitates elimination of harmful agents, repair of damaged structures, and restoration of homeostasis. Inflammation may be acute or chronic in nature. Acute inflammation develops within minutes to hours, typically persists for several days, and is initiated primarily by tissue-resident macrophages and dendritic cells. In response to stimuli perceived as harmful, these cells release cascades of pro-inflammatory cytokines, chemokines, and prostaglandins, including prostaglandin E2 (PGE2). Acute inflammation is characterized by three main stages: increased blood flow to the affected area due to vasodilation of small vessels, enhanced vascular permeability, and migration of phagocytic leukocytes into the affected tissue. A properly resolved acute inflammatory response leads to elimination of pathogens or necrotic cells, followed by tissue repair. However, leukocytes can also contribute to collateral damage of healthy tissues during physiological inflammatory responses. When acute inflammation fails to resolve due to persistent tissue injury or dysregulation of regulatory mechanisms, chronic inflammation develops [19].

Chronic inflammation is increasingly recognized as a major contributor to the development and progression of non-communicable diseases (NCDs), driven by sustained overproduction of pro-inflammatory mediators such as reactive oxygen species, eicosanoids, cytokines, and chemokines [20]. Persistent inflammatory activity can lead to tissue damage and dysfunction, impairment of barrier integrity, and infiltration of inflammatory cells, often resulting from immune dysregulation or prolonged exposure to irritants. These processes are further amplified by elevated levels of high-sensitivity C-reactive protein (hs-CRP), interleukin-6 (IL-6), and tumor necrosis factor alpha (TNF-α), which are strongly associated with major NCDs [21,22]. In individuals with visceral obesity and metabolic syndrome, chronic low-grade inflammation, often referred to as “metaflammation”, is closely linked to adipose tissue dysfunction. Adipocyte hypertrophy, hypoxia, and extracellular matrix remodelling promote oxidative stress and endoplasmic reticulum stress, leading to increased chemokine production and recruitment of circulating monocytes. These monocytes differentiate into pro-inflammatory M1 macrophages that accumulate around dead or dying adipocytes, forming so-called crown-like structures [23]. Recent transcriptomic analyses provide additional support for this phenotypic transition. RNA-sequencing studies of adipose tissue macrophages demonstrate a shift from alternatively activated anti-inflammatory M2 macrophages toward a pro-inflammatory M1 transcriptional program in obesity. This shift is characterized by increased expression of genes involved in cytokine production and inflammasome signaling, accompanied by downregulation of genes associated with tissue repair and anti-inflammatory responses, confirming that macrophage polarization reflects coordinated transcriptomic reprogramming contributing to metaflammation and metabolic dysfunction [24]. M1 macrophages and dysfunctional adipocytes secrete numerous adipokines and inflammatory mediators, including TNF-α, IL-6, interleukin-1β (IL-1β), resistin, and free fatty acids, thereby activating signalling pathways involving Toll-like receptor 4 (TLR4), nuclear factor κB (NF-κB), and c-Jun N-terminal kinase (JNK) in the liver, skeletal muscle, and pancreatic β-cells. These processes exacerbate insulin resistance, impair insulin signalling, and contribute to the development of MetS components, including hyperglycemia, atherogenic dyslipidaemia, and hypertension [23,25,26]. Increasing evidence also highlights the key role of the NOD-like family receptor inflammasome, containing pyrin domain 3 (NLRP3) as a link between nutrient excess and chronic inflammation in obesity and MetS. Excess saturated fatty acids, cholesterol crystals, reactive oxygen species, and fluctuations in blood glucose levels activate the NLRP3 complex in adipose tissue macrophages and pancreatic β-cells, leading to maturation of interleukin-1β (IL-1β) and interleukin-18 (IL-18), thereby promoting insulin resistance and β-cell dysfunction [26,27]. In parallel, Western-type diets rich in saturated fats and poor in fermentable fibre may induce dysbiosis, reduce short-chain fatty acid production, and impair tight-junction integrity, thereby facilitating translocation of lipopolysaccharide (LPS) from Gram-negative bacteria into the circulation. This phenomenon, referred to as metabolic endotoxemia, further amplifies TLR4/NF-κB signaling and provides an additional mechanistic link between diet-induced dysbiosis, gut barrier dysfunction, and chronic low-grade inflammation in MetS [2,8].

In individuals with obesity and MetS, chronic low-grade inflammation, insulin resistance, and atherogenic dyslipidaemia collectively increase the risk of several chronic inflammatory diseases, particularly cardiovascular diseases (CVD). Atherosclerosis represents a chronic inflammatory disease of the arterial wall in which endothelial dysfunction promotes monocyte recruitment, macrophage activation, and foam-cell formation following uptake of oxidized low-density lipoprotein (oxLDL). Persistent inflammatory signaling and extracellular matrix remodelling contribute to plaque progression and instability, linking metabolic syndrome with increased risk of myocardial infarction, stroke, and other cardiovascular complications [28].

## 3. Anti-Inflammatory Diet in the Management of Metabolic Syndrome

### 3.1. Dietary Models and Inflammatory Potential

In the literature, the most frequently described dietary patterns with anti-inflammatory potential in the context of metabolic syndrome (MetS) include the Dietary Approaches to Stop Hypertension (DASH) diet, the Mediterranean diet (MedDiet), and low-glycemic index (LGI) dietary models. These dietary patterns share several key characteristics, including a high intake of plant-based foods, dietary fibre, antioxidants, and unsaturated fatty acids, alongside reduced consumption of saturated fats, simple sugars, and ultra-processed foods. Collectively, these features contribute to attenuation of chronic inflammation and improvement of metabolic parameters. Importantly, the practical applicability of these diets is enhanced by their reliance on simple food-based principles, flexibility, and adaptability to individual dietary preferences without the need for highly restrictive rules, making them suitable for long-term adherence [29,30,31].

The DASH diet emphasizes whole grains, fruits, vegetables, legumes, low-fat dairy products, lean poultry, and fish, while limiting saturated fats, cholesterol, red meat, refined grains, and sweets [32,33]. The dietary components characteristic of the DASH pattern are strongly associated with anti-inflammatory dietary profiles [34]. Intervention studies have demonstrated that adherence to the DASH diet results in significant reductions in blood pressure, improvements in carbohydrate and lipid metabolism, and decreased levels of inflammatory markers in individuals with overweight, obesity, and MetS [35,36].

Numerous studies have also investigated the Mediterranean diet. For example, the PREDIMED trial reported a 40% reduction in the incidence of type 2 diabetes mellitus (T2DM) in the intervention group following a Mediterranean diet supplemented with extra-virgin olive oil compared with a low-fat diet. Moreover, a recent meta-analysis of 50 prospective and randomized controlled trials demonstrated a positive association between adherence to the MedDiet and a 50% reduction in the prevalence of MetS [37]. Evidence further suggests that the MedDiet influences epigenomic regulation and metabolic homeostasis. Both short- and long-term adherence to this dietary pattern has been associated with alterations in microRNA (miRNA) expression profiles and transcriptional regulation of genes involved in inflammatory, adipogenic, and atherogenic pathways implicated in MetS pathogenesis [38]. Consistent with these findings, Marques-Rocha et al. [39] reported altered expression of miRNAs associated with cardiovascular disease pathogenesis in patients with MetS following an 8-week hypocaloric Mediterranean diet intervention.

The low-glycemic index (LGI) diet represents another dietary approach with documented anti-inflammatory effects in individuals with obesity and MetS. This dietary pattern is characterized by a high intake of complex carbohydrates and dietary fibre, including whole grains, legumes, vegetables, and low-glycemic index fruits, while limiting refined carbohydrates and simple sugars [40]. Randomized clinical trials have shown that LGI or low-glycemic load (LGL) diets reduce levels of hs-CRP and selected pro-inflammatory cytokines such as IL-6, and improve adipokine profiles, including increased adiponectin concentrations, in individuals with overweight and obesity, including those with MetS [41,42,43]. Notably, some studies have demonstrated that anti-inflammatory effects persist after adjustment for weight loss, suggesting that carbohydrate quality (LGI/LGL), rather than weight reduction alone, plays a significant role in modulating chronic inflammation [39,40]. Meta-analyses further confirm that long-term adherence to LGI/LGL dietary patterns is associated with reduced CRP levels and improvements in carbohydrate and lipid metabolism, which are particularly relevant for the prevention and management of MetS [40,44].

One of the earliest clinical applications of an “anti-inflammatory diet” in a patient cohort was reported around 2003 in studies involving individuals with rheumatoid arthritis. Early dietary interventions consisted of reduced intake of arachidonic acid combined with fish oil supplementation and demonstrated significant reductions in disease activity and inflammatory biomarkers [45]. One of the earliest explicit uses of the term “anti-inflammatory diet” in the title of a scientific publication is attributed to Barry Sears in “The Zone Diet: An Anti-Inflammatory, Low Glycemic-Load Diet” [46] which proposed a structured dietary model characterized by a low glycemic load, balanced macronutrient distribution, and increased intake of omega-3 fatty acids. In subsequent years, the concept of anti-inflammatory dietary patterns was further explored in interventional research, including studies examining their supportive role in the management of inflammatory bowel diseases [47]. Around 2014, a marked increase in scientific publications addressing anti-inflammatory diets was observed, reflecting rapidly growing research interest in this dietary approach (reported increase of approximately 2475% between 2014 and 2025) [48]. Concurrently, several review articles synthesized common characteristics of dietary patterns described as anti-inflammatory, highlighting substantial overlap with the Mediterranean, DASH, and low-glycemic index (LGI) diets [49]. A defining feature of anti-inflammatory dietary strategies is the emphasis on modifying fatty acid composition, particularly increasing the intake of omega-3 polyunsaturated fatty acids (PUFAs).

### 3.2. Practical Nutritional Characteristics of an Anti-Inflammatory Diet

Omega-3 (n-3) and omega-6 (n-6) polyunsaturated fatty acids are essential dietary components that cannot be synthesized by the human body itself. While both classes of PUFAs positively affect health conditions, omega-3 fatty acids exert more pronounced anti-inflammatory and health-promoting benefits. Maintaining a dietary balance favouring omega-3 intake has been associated with improved metabolic health and increased longevity [50,51]. Alpha-linolenic acid (ALA; 18:3 n-3) is a plant-derived omega-3 fatty acid that can undergo elongation and desaturation to form long-chain omega-3 fatty acids, including eicosapentaenoic acid (EPA; 20:5 n-3) and, to a lesser extent, docosahexaenoic acid (DHA; 22:6 n-3). These metabolic pathways involve the same enzymatic systems responsible for omega-6 metabolism, particularly delta-6 desaturase (D6D) and delta-5 desaturase (D5D). Through these pathways, ALA is converted into successive metabolites (e.g., stearidonic acid and EPA), while linoleic acid (LA; 18:2 n-6) is metabolized into arachidonic acid (AA; 20:4 n-6). Consequently, ALA and LA compete for the same enzymatic pathways, meaning that higher ALA intake may inhibit omega-6 metabolism. Increased ALA consumption has been associated with reduced production of omega-6-derived metabolites, including decreased AA levels (and its precursors), accompanied by increased concentrations of unmetabolized LA. This phenomenon is explained by the higher affinity of D6D for omega-3 fatty acids compared with omega-6 fatty acids. In practical terms, excess ALA occupies shared enzymatic pathways, thereby limiting conversion of LA into pro-inflammatory AA and its downstream eicosanoids. Furthermore, dihomo-γ-linolenic acid (DGLA; 20:3 n-6) and EPA compete with AA for cyclooxygenase and lipoxygenase enzymes, resulting in the production of eicosanoids with less pro-inflammatory activity, for example series-1 prostaglandins derived from DGLA and series-3 prostaglandins derived from EPA, rather than the more pro-inflammatory series-2 compounds originating from AA [52].

Evidence from large epidemiological cohorts supports the importance of fatty acid balance. In a study of more than 85,000 participants from the UK Biobank followed for approximately 13 years, individuals with the highest omega-6 to omega-3 fatty acid ratios had a 26% higher risk of all-cause mortality, a 14% higher risk of cancer mortality, and a 31% higher risk of cardiovascular mortality compared with those with the lowest ratios [53]. Western dietary patterns typically demonstrate omega-6 to omega-3 ratios of approximately 15–16.7:1 [54], reflecting insufficient omega-3 intake and excessive omega-6 consumption compared with the estimated ancestral dietary ratio of approximately 1:1. In secondary prevention of cardiovascular disease, an omega-6 to omega-3 ratio of 4:1 has been associated with a 70% reduction in total mortality, whereas ratios of 2–3:1 have been shown to reduce inflammatory activity in patients with rheumatoid arthritis [54]. Diets rich in ALA have also been shown to be effective in the secondary prevention of coronary events and cardiovascular mortality [55]. To achieve a more favorable fatty acid balance, replacement of certain commonly used plant oils high in saturated fats or omega-6 fatty acids, such as coconut oil, sunflower oil, or peanut oil, with plant oils rich in omega-3 fatty acids is recommended. Examples include camelina oil (Camelina sativa; approximately 38% ALA), flaxseed oil (Linum usitatissimum; approximately 55% ALA), and chia oil (Salvia hispanica; approximately 61% ALA) [56]. Such substitutions in individuals with metabolic syndrome may contribute to reductions in inflammatory markers, including decreased secretion of interleukin-6 (IL-6) [57]. Incorporating ground flaxseed and chia seeds into the diet is also beneficial, not only because of their high ALA content but also due to their fibre and antioxidant compounds. Lignans present in flaxseed are metabolized by the gut microbiota into biologically active enterolignans with anti-inflammatory potential. These compounds may additionally influence epigenetic mechanisms, including DNA methylation and histone modification, thereby contributing to alterations in gene expression associated with inflammation and oxidative stress [58,59].

A diet rich in fats, refined carbohydrates, and processed sources of animal protein is commonly associated with elevated levels of inflammation, whereas dietary patterns rich in fruits, vegetables, and fish are associated with lower levels of inflammation or even anti-inflammatory profiles [60,61,62]. Therefore, dietary patterns influence the regulation of chronic inflammation, establishing diet as a key factor affecting inflammatory status in individuals with obesity [63,64]. In individuals with metabolic syndrome (MetS), combining regular physical activity with a specialized dietary approach is essential for improving health outcomes. Such a diet should limit sources of saturated fatty acids and trans fats, provide anti-inflammatory omega-3 fatty acids, and include low-glycemic index foods, whole-grain cereal products, polyphenols and nuts. Processed carbohydrate sources should be restricted [65]. An anti-inflammatory dietary pattern should also include low-glycemic index fruits and vegetables rich in polyphenols, including flavonoids, which exert beneficial effects through antioxidant activity and reduction in inflammatory processes. Representative examples include berries such as aronia, blackcurrants, blueberries, raspberries, and strawberries, as well as vegetables including red onion, spinach, red cabbage, broccoli, kale, and red bell pepper [66,67]. Implementation of these dietary recommendations has been associated with reduced incidence of ischemic heart disease, decreased levels of inflammation, reduced production of reactive oxygen species, decreased secretion of pro-inflammatory cytokines such as IL-6, IL-18, and TNF-α, and increased production of anti-inflammatory cytokines [65]. Interdisciplinary targeted interventions conducted in individuals with MetS have demonstrated reductions in associated metabolic abnormalities, including fasting glycemia, waist circumference, systolic and diastolic blood pressure, and triglyceride concentrations [68]. In a meta-analysis comparing low-carbohydrate diets with low-fat diets (defined as <30% of daily energy intake from fat), researchers confirmed that both dietary interventions produced beneficial effects on insulin resistance and reduced levels of CRP, IL-6, and TNF-α. A particularly important determinant of improvement was the magnitude of weight loss achieved (ranging from 0.7 to 14 kg). Greater reductions in adipose tissue were associated with greater reductions in inflammatory markers [69]. The relationship between weight loss and inflammatory marker levels may be explained by the fact that adipose tissue represents a major source of pro-inflammatory cytokines such as IL-6 and TNF-α, which promote hepatic lipogenesis [70,71] and trigger a systemic acute-phase response [72].

Concurrently, an increasing number of studies employ omics-based approaches to analyse the effects of diet on key pathophysiological pathways involved in obesity and metabolic syndrome. Precision nutrition research indicates that integrating genomic, epigenomic, transcriptomic, metabolomic, and metagenomic data can improve understanding of the heterogeneous patient response to dietary interventions [73,74]. Interventional dietary studies, including weight reduction programs based on the Mediterranean diet and very-low-calorie ketogenic diets, using multi-omics methodologies have documented simultaneous changes in gut microbiota composition, miRNA expression profiles, and DNA methylation patterns, which are associated with improvements in metabolic parameters and reductions in inflammatory status [39,75,76].

Integration of omics data may facilitate the development of more personalized nutritional therapies aimed at preventing chronic diseases and optimizing individual responses to dietary interventions [77]. Multi-omics research increasingly relies on computational strategies that integrate heterogeneous datasets derived from metagenomics, transcriptomics, metabolomics, and epigenomics. Bioinformatic frameworks and machine-learning algorithms enable the identification of molecular signatures linking dietary exposures with metabolic phenotypes [17,18]. Integrative tools such as Multi-Omics Factor Analysis (MOFA) and DIABLO frameworks within the mixOmics platform allow coordinated analysis of multiple biological layers and facilitate the discovery of pathways that cannot be detected through single-omics approaches [17,78]. In the context of metabolic syndrome, such integrative analyses have revealed interactions between microbiota-derived metabolites, host epigenetic regulation, and transcriptomic control of inflammatory and metabolic genes. Consequently, multi-omics integration represents a key methodological foundation for precision nutrition research, supporting the concept that dietary components can act as molecular modulators of metabolic pathways across interconnected biological systems [18]. Subsequent subsections present selected examples of multi-omics diet-related interactions with gut microbiota, DNA methylation, and adipokine profiles. At the same time, the strength of evidence differs across these layers: clinical and interventional data are relatively strongest for improvements in inflammatory biomarkers, insulin sensitivity, and selected adipokines after anti-inflammatory dietary patterns, whereas evidence linking diet to microbiota-derived metabolites and DNA methylation in MetS is often based on smaller human studies supported by mechanistic animal or in vitro data. Therefore, the integrated multi-omics model should be interpreted as a biologically plausible framework with uneven levels of validation across individual pathways [7,8].

## 4. Relationship Between an Anti-Inflammatory Diet, the Microbiome, and Metabolic Syndrome

Diet can be considered one of the most important factors shaping microbiota composition, explaining approximately 20–50% of its variability in humans [79,80,81]. A Western-type diet, characterized by a high intake of saturated fats and simple sugars and a low intake of dietary fibre, has been consistently associated with reduced gut microbiota diversity and an increased abundance of bacteria linked to activation of pro-inflammatory responses [79,82]. A high-fat diet rich in saturated fatty acids (SFA) leads to dysbiosis, including a reduction in beneficial bacteria such as *Roseburia* spp. [83,84]. Diet-induced metabolic alterations contribute to modifications in gut microbiota composition [85,86], promoting the growth of Gram-negative bacteria such as Enterobacteriaceae, which results in increased lipopolysaccharide (LPS) production [87,88]. Consequently, this leads to chronic low-grade inflammation, elevated plasma inflammatory markers, and impaired intestinal barrier permeability [89,90]. Disruption of gut barrier integrity may result in chronic systemic inflammation, organ dysfunction, and the development of metabolic diseases [91]. Chronic low-grade inflammation accompanied by insulin resistance constitutes a key element in the pathophysiology of metabolic syndrome (MetS). Gut bacteria and their components, including endotoxins, may translocate into the bloodstream due to microbiota imbalance and compromised intestinal barrier function, triggering systemic inflammatory responses [92,93]. The Western diet is a significant contributor to chronic endotoxemia, defined as an excess of circulating bacterial cell wall components resulting from impaired intestinal barrier function and microbiota alterations. Endotoxemia represents a major mechanism linking the gut microbiota with metabolic syndrome and systemic low-grade inflammation [82].

Saturated fats, present for example in dairy products, promote the growth of delta-Proteobacteria such as *Bilophila wadsworthia*. The expansion of *B. wadsworthia* has been linked to impaired intestinal homeostasis, disruption of the mucosal barrier, and promotion of pro-inflammatory immune responses, thereby contributing to intestinal and systemic inflammation [94]. Conversely, a diet rich in safflower oil (high in omega-6 PUFA) has been associated with reduced Bacteroides abundance and increased proportions of Firmicutes, Actinobacteria, and Proteobacteria. Such diet-induced shifts in gut microbiota may favor dysbiosis, increased intestinal permeability, and enhanced inflammatory signaling; however, these effects depend on the specific taxa expanded within these phyla rather than on phylum-level changes alone [95]. Habitual intake of monounsaturated fatty acids (MUFA) and polyunsaturated fatty acids (PUFA) has been associated with differences in the abundance of Bacteroides spp. and Bifidobacterium spp.; however, the direction and magnitude of these associations appear to depend on the overall dietary pattern and the specific fatty acid profile consumed. Moreover, an imbalance in dietary fatty acid composition, particularly in the n-6/n-3 PUFA ratio, may influence immune responses, including Th1/Th2 activity and cytokine production [96,97]. Regular consumption of red meat, rich in trans and saturated fats, also increases the abundance of Bacteroides species [98].

In contrast, omega-3 fatty acids exert beneficial effects on gut microbiota. Numerous studies indicate that the inclusion of omega-3 fatty acids (EPA/DHA) in the diet may modulate microbiota composition and support intestinal barrier integrity by influencing epithelial membrane structure and tight-junction pathways, thereby potentially reducing intestinal permeability and bacterial pro-inflammatory signals [97,99]. In this context, plant-based sources of omega-3 fatty acids such as alpha-linolenic acid (ALA), including chia seeds and flaxseed, should also be emphasized. These foods provide important sources of dietary fibre and bioactive compounds capable of modulating microbiota composition and intestinal barrier function [100,101]. An anti-inflammatory diet should additionally include walnuts, which are a valuable source of ALA (approximately 10% of total fatty acids), fibre, and bioactive antioxidant and anti-inflammatory compounds [102]. Examples of such compounds include ellagitannins (ET) and ellagic acid (EA), polyphenols present in pomegranate, walnuts, and selected berries. A substantial proportion of ET and EA undergoes metabolic transformation within the gastrointestinal tract, particularly in the colon, where gut microbiota convert them into urolithins (Uros) [103,104,105]. Urolithin A (Uro-A) is one of the principal metabolites of ET/EA and exhibits a broad spectrum of biological activities, including antioxidant, anti-inflammatory, neuroprotective, antidiabetic, and anticancer effects [106,107]. Importantly, the capacity to produce Uro-A varies between individuals and depends on microbiota composition (so-called “metabotypes”); therefore, the health effects of ET/EA consumption may differ between individuals even when intake levels of the same foods are comparable [107].

A low-glycemic index (LGI) diet is characterized by a high intake of dietary fibre and complex carbohydrates rich in starch, the consumption of which leads to gradual glucose release into the bloodstream and reduced insulin secretion [108]. Intake of LGI foods has been associated with higher HDL cholesterol levels, reductions in total and LDL cholesterol, decreased visceral adiposity, and a lower risk of developing type 2 diabetes mellitus (T2DM) and cardiovascular disease (CVD) [108,109,110]. A diet rich in soluble fibre and complex carbohydrates promotes the growth of beneficial gut bacteria that support proper gastrointestinal function, nutrient metabolism, and immune system activity. Collectively, these mechanisms may contribute to the prevention of non-communicable diseases such as obesity, T2DM, and metabolic syndrome (MetS).

High intake of soluble fibre increases the abundance of Bacteroides spp., the Clostridium leptum group, and Eubacterium rectale [96]. Bacteroides spp. are important commensal bacteria involved in the degradation of complex carbohydrates and in the generation of microbial metabolites in the colon. Their increased abundance in response to fibre-rich diets is generally considered beneficial, although their biological effects remain species-dependent. Soluble fibres are particularly associated with an increase in butyrate-producing bacteria, especially those within the Clostridium leptum group and Eubacterium rectale [111]. Complex carbohydrates also promote the proliferation of *Bifidobacterium* spp., including *B. longum* and *B. breve*, as well as saccharolytic bacteria such as Bacteroides thetaiotaomicron [112]. Dietary fibre stimulates bacterial fermentation in the colon, resulting in increased production of short-chain fatty acids (SCFA) such as acetate and butyrate, which in turn enhances the synthesis and secretion of glucagon-like peptide-1 (GLP-1)—a satiety hormone with a central role in appetite regulation and the pathophysiology of obesity. GLP-1 is not produced by the microbiota itself but by intestinal L cells; however, microbial metabolites (particularly SCFA and secondary bile acids) may significantly enhance its secretion. Therefore, diets that modulate the microbiota indirectly influence GLP-1 levels. The impact of dietary patterns on bacterial fermentation and subsequent GLP-1 synthesis has been demonstrated in a one-year intervention involving high wheat-fibre intake (24 g/day) among individuals with hyperinsulinemia; after nine months, acetate and butyrate concentrations increased, and after twelve months, GLP-1 levels rose by approximately 25% compared with baseline and were higher than in the control group. [113]. Incorporation of vinegar into the diet, particularly apple cider vinegar, may also be beneficial, as it provides exogenous acetate—an SCFA whose administration in obese mice has been shown to reduce food intake, inhibit body weight gain, and decrease insulin resistance [114]. In patients with obesity and MetS, peppers (both as vegetables and spices) may offer multi-omics benefits due to their polyphenol content and, in spicy varieties, capsaicin. Consumption has been associated with increased Faecalibacterium abundance (linked to higher butyrate levels) and concurrent modulation of gut signalling pathways, including the TRPV1–incretin hormone axis (e.g., GLP-1). Dietary inclusion of capsaicin-rich peppers may also beneficially influence bacteria involved in intestinal barrier integrity and mucus production, partly through effects on Akkermansia populations. However, appropriate dosing of capsaicin is essential, as excessive intake may produce adverse effects due to the irritant properties of this alkaloid [115].

Legumes represent a particularly valuable component of a low-GI diet because they provide complex carbohydrates, resistant starch, and fermentable fibre. The use of beans in dietary therapy for obesity has been associated with reductions in body weight through enhanced satiety and microbiota-mediated mechanisms. Regular consumption promotes favourable shifts in microbiota composition and gut metabolite profiles, potentially supporting intestinal barrier function and reducing pro-inflammatory signals associated with dysbiosis. In an intervention study involving individuals with obesity, bean consumption was associated with microbiome and metabolite changes toward a profile more conducive to metabolic health, highlighting the potential of legumes as a dietary therapy tool in high-risk populations. Therefore, legumes may serve as a practical “link” between LGI dietary patterns and gut microbiota modulation in obesity and MetS [116,117]. Similarly, raspberries and strawberries, fruits rich in dietary fibre and polyphenols and characterized by low energy density, may support the growth of metabolically beneficial bacteria by providing fermentable substrates and promoting the production of microbiota-derived metabolites associated with intestinal barrier function. Supplementation with red raspberries at a daily portion of 125 g has been shown to modulate microbiome composition and gut metabolite profiles toward a potentially favourable metabolic health phenotype, including a twofold increase in Eubacterium eligens and a 60% reduction in Ruminococcus gnavus. This intervention also reduced insulin resistance and cholesterol levels, particularly LDL-C [118]. Dietary interventions based on strawberries have likewise been associated with increased microbiota diversity and enrichment of SCFA-producing bacteria [119].

Fermented vegetables (e.g., unpasteurized sauerkraut and traditionally lacto-fermented cucumbers) may further strengthen microbiota-targeted dietary patterns by combining fermentable plant fibre with live lactic acid bacteria and fermentation-derived metabolites; in a controlled human intervention, higher fermented-food intake increased gut microbial diversity and reduced multiple inflammatory markers [120]. Notably, red cabbage is characterized by particularly high polyphenol content (largely driven by anthocyanins), substantially exceeding that of white cabbage, which makes it a valuable raw material for fermented-vegetable interventions focused on phytochemical delivery [121]. Moreover, fermentation can enhance the functional availability of phytochemicals: during sauerkraut fermentation, LAB succession has been linked to an increased pool of total free phenolics (including free phenolic acids), consistent with a shift toward forms that may be more bioactive [122]. The relationship between foods that stimulate microbiota activity and the synthesis of bioactive metabolites provides a natural bridge to epigenetics, as certain polyphenol metabolites and their derivatives may influence gene expression through modulation of DNA methylation and epigenetic enzyme activity [123].

To strengthen the mechanistic bridge between metabolomics and epigenetics, it should be emphasized that microbiota-derived SCFAs (e.g., acetate and butyrate) act not only as energy substrates but also as signaling molecules and epigenetic regulators. SCFAs function as ligands for G-protein coupled receptors GPR41 (FFAR3) and GPR43 (FFAR2), thereby modulating immune and metabolic pathways, while butyrate and related SCFAs can inhibit histone deacetylases (HDACs), promoting histone acetylation and a more “open” chromatin state that favors transcription of anti-inflammatory and cytoprotective genes. This dual receptor–epigenetic mechanism provides a concrete example of how diet-derived microbial metabolites can act as ‘epi-drugs’ at the molecular level [124].

## 5. Anti-Inflammatory Diet and DNA Methylation

Aging cannot be prevented; however, age-related changes in DNA methylation that may contribute to the development of age-associated disorders can potentially be limited or delayed. Aging is accompanied by significant genome-wide alterations in DNA methylation patterns, including global hypomethylation and localized hypermethylation of selected gene promoters. Emerging evidence suggests that the loss of epigenetic information may represent a reversible driver of aging processes [125]. Biological pathway analyses indicate that age- and BMI-associated methylation changes are overrepresented among genes involved in cancer development, T2DM, and CVD. This is further supported by recent findings showing that both epigenetic and polygenic factors contribute to BMI variability, and that DNA methylation-based models may improve the prediction of obesity-related traits. These observations reinforce the relevance of methylation signatures as potential biomarkers of metabolic risk and obesity-associated biological alterations [126]. Moreover, epigenetic biomarkers measured in blood may partially reflect DNA methylation changes occurring in target tissues associated with metabolic diseases such as obesity [127,128]. Importantly, despite exposure to factors that accelerate epigenetic aging, lifestyle modifications, including smoking cessation, increased physical activity, weight reduction, and improved sleep hygiene, may promote beneficial DNA methylation changes and reduce biological age [129,130,131].

DNA methylation processes are also closely linked to dietary patterns. Bioactive food components and dietary habits exert beneficial effects on key hallmarks of aging, including oxidative stress, mitochondrial function, apoptosis and autophagy, genome stability, and immune function [132]. Anti-inflammatory dietary interventions, particularly the Mediterranean diet, characterized by high intake of omega-3 fatty acids, polyphenols, and whole-grain products, have been associated with reductions in biological age, with more pronounced effects observed in individuals classified as “epigenetically older” [133]. Studies have demonstrated a positive correlation between methylation of the EEF2 gene, which encodes eukaryotic elongation factor 2, a protein involved in the elongation step of mRNA translation during protein synthesis, and concentrations of TNF-α and CRP, suggesting that epigenetic variation in this gene may be associated with inflammatory status. Thus, although EEF2 is not a classical inflammatory marker itself, its methylation status may reflect inflammation-related regulatory changes linked to diet and biological aging [134]. Moreover, adherence to the Mediterranean diet (MedDiet) has been linked to changes in methylation levels of inflammation-related genes, suggesting a potential regulatory role [135]. Higher diet quality has been inversely associated with biological age. Using DASH diet recommendations as an indicator of high dietary quality [28], researchers observed slower epigenetic aging and reduced risk of disease and mortality, with more pronounced effects among individuals with low physical activity levels [136]. Polyphenols represent key bioactive dietary components influencing DNA methylation. Curcumin, a polyphenolic compound found in turmeric, is among the most potent dietary polyphenols and exhibits numerous molecular targets, contributing to its broad therapeutic potential. Evidence suggests that curcumin may support the management of inflammatory conditions, metabolic syndrome, pain, degenerative eye diseases, and kidney disorders, primarily due to its antioxidant and anti-inflammatory properties [137]. When incorporating turmeric into the diet, combining it with black pepper is advisable, as piperine synergistically enhances antioxidant potential; together, they have been shown to produce a 27-fold greater reduction in malondialdehyde (MDA) levels [138]. In addition to polyphenols, organosulfur compounds found in garlic also influence epigenetic pathways, including DNA methylation. Studies have identified diallyl trisulfide (DATS), accounting for over 40% of garlic’s total composition, as a bioactive compound capable of enhancing CpG demethylation or promoting histone acetylation as an epigenetic modulator. Furthermore, DATS inhibited MSU-induced IL-1β secretion and caspase-1 activation, reduced inflammasome complex formation in macrophages, restored mitochondrial function, and decreased MSU-induced NOX3/4 expression, demonstrating anti-inflammatory and anticancer properties [139,140].

The increasing prevalence of obesity and metabolic dysfunction is partly associated with higher consumption of energy-dense foods rich in fats and simple sugars. Such dietary patterns may exacerbate inflammation through epigenetic mechanisms [141]. Elevated glucose concentrations influence gene expression in human pancreatic islets, with some genes simultaneously exhibiting epigenetic alterations that may contribute to impaired insulin secretion observed in T2DM [142]. Dietary fat quality may epigenetically remodel adipose tissue rather than acting solely through caloric excess. Short-term high-fat overfeeding can alter DNA methylation patterns in subcutaneous adipose tissue [143]. In a human overfeeding trial with similar weight gain between groups, excess SFA intake induced greater hepatic fat accumulation and ~twofold higher increases in visceral adipose tissue (VAT) than PUFA, whereas PUFA overfeeding was associated with threefold larger gains in lean body mass. Hepatic fat gain correlated positively with plasma SFA and inversely with PUFA, alongside distinct SAT gene-regulation patterns linked to energy storage and insulin resistance [144]. Moreover, experimental studies suggest that specific fatty acids can modify promoter methylation of inflammatory and adipose-remodeling genes, e.g., TNF-α, Vascular Endothelial Growth Factor B (VEGFB), supporting a methylation-mediated pathway through which SFA and PUFA influence adipose endocrine and inflammatory signaling [145]. In patients with obesity and metabolic disorders, omega-3 PUFA appear to exert a particularly important influence on the epigenetic regulation of inflammation [146]. Due to their capacity to correct abnormal acetylation and methylation profiles in various non-communicable diseases, omega-3 fatty acids have been identified as potential “epi-drugs” [147]. Based on current evidence, achieving beneficial epigenetic effects may involve reducing red meat consumption, replacing it with lean poultry or, preferably, fish rich in omega-3 fatty acids [148,149], and limiting overall dietary energy intake [131].

Caloric restriction has likewise been associated with favourable epigenetic modifications in DNA methylation processes [150]. Among individuals with low birth weight (LBW), who face increased risks of insulin resistance, T2DM, and obesity in adulthood, fasting interventions may modulate the activity of genes encoding key adipokines, including ADIPOQ, responsible for adiponectin secretion, and LEP, responsible for leptin production [151,152]. Gene activity is influenced by epigenetic mechanisms, including DNA methylation [151]. Functionally, changes in promoter-region methylation may affect transcription: hypomethylation is associated with increased gene expression, whereas hypermethylation is linked to gene repression [153]. Importantly, epigenetic regulation of the ADIPOQ gene has been identified as a key molecular mechanism linking obesity with hypoadiponectinemia. Studies indicate that obesity-associated metabolic stress may promote hypermethylation within regulatory regions of the ADIPOQ promoter in adipose tissue, which suppresses adiponectin gene transcription and contributes to insulin resistance and metabolic dysfunction. This epigenetic repression provides a mechanistic explanation for the reduced circulating adiponectin concentrations frequently observed in individuals with metabolic syndrome [153]. Consequently, fasting may potentially increase adiponectin production and decrease leptin secretion, although short-term interventions do not always translate directly into measurable gene expression changes [154]. Adiponectin and leptin are adipokines involved in regulating glucose and lipid metabolism as well as satiety, and their secretion is often dysregulated in obesity [155,156]. Analysis of adipokine levels in relation to specific dietary modifications represents an important component in evaluating the effectiveness of dietary interventions among patients with metabolic syndrome (MetS). These findings suggest that diet-induced epigenetic modifications may directly influence adipokine secretion profiles, thereby linking nutritional factors with adipose tissue endocrine function and systemic metabolic regulation.

## 6. Anti-Inflammatory Diet and Adipokine Levels

Adipose tissue is an active endocrine organ that regulates systemic homeostasis through the secretion of adipokines. In obesity, pro-inflammatory immune cells infiltrate adipose tissue, leading to dysregulated adipokine secretion, metabolic disturbances, and the development of chronic low-grade inflammation “metaflammation” [22]. A shift in the secretory profile of adipose tissue toward pro-inflammatory and proatherogenic adipokines is reflected by increased synthesis of leptin, resistin, omentin, visfatin, TNF-α, and IL-6, along with reduced production of anti-inflammatory adipokines such as adiponectin and IL-10 [157,158,159]. Among the best-characterized adipokines with significant clinical relevance are leptin and adiponectin. Leptin is involved in regulating satiety, energy homeostasis, and immune function [160]. In obesity, leptin resistance is frequently observed, and leptin itself may act as a pro-inflammatory mediator, contributing to metabolic disturbances such as insulin resistance and CVD [161]. In contrast, adiponectin exerts protective effects across various physiological processes, including energy metabolism, inflammation regulation, and cell proliferation, and has demonstrated protective properties in CVD [162]. The inverse correlation between adiponectin levels and metabolic diseases suggests that adiponectin may serve as a non-invasive biomarker of disease status. Strategies aimed at increasing adiponectin secretion may represent an effective approach in the prevention and treatment of conditions associated with hypoadiponectinemia, such as obesity and T2DM [163]. The ADIPO/LEP index has been introduced as a marker of adipose tissue dysfunction, reflecting the pathophysiology of adipokine secretion [164]. Notably, this index decreases with an increasing number of metabolic syndrome risk factors [165].

Dietary modification represents the primary intervention in the management of metabolic syndrome and regulation of adipokine profiles. Nutritional changes appear to exert a stronger influence than exercise alone on beneficial alterations in adiponectin and leptin secretion [166]. Favourable effects on adiponectin concentrations have been observed with the Mediterranean diet, DASH diet, plant-based dietary patterns, and energy-restricted diets [167]. Specific foods and nutrients known to increase adiponectin levels include monounsaturated fatty acids (MUFA), omega-3 PUFA, dietary fibre, polyphenols, and dairy products. In contrast, components typical of a Western dietary pattern, such as SFA, trans fatty acids, simple sugars and disaccharides, and red meat, negatively affect adiponectin levels. Diets characterized by a high glycemic index are also associated with reduced adiponectin concentrations [167]. Combining structured physical training with a high-protein, low-glycemic index (LGI) diet over six weeks has been shown to increase satiety, promote a negative energy balance, and enhance adiponectin secretion, while simultaneously reducing pro-inflammatory markers such as IL-6 and hs-CRP [41]. Such interventions have also been associated with reductions in circulating leptin, asprosin, and irisin, contributing to improvements in metabolic syndrome parameters [109,110]. Importantly, combining targeted dietary strategies with aerobic-resistance training appears essential, as physical activity alone may not increase adiponectin synthesis even after 12 weeks of aerobic or combined training programs [168]. Supplementation with omega-3 fatty acids has also demonstrated beneficial effects on circulating adiponectin levels, particularly when administered at doses exceeding 2 g/day for at least 10 weeks; however, such supplementation does not appear to significantly alter serum leptin concentrations [169]. Omega-3 supplementation is additionally associated with reductions in systemic inflammation, reflected by decreased CRP levels and lower concentrations of pro-inflammatory adipokines such as IL-6 and TNF-α [170,171].

## 7. Limitations in the Application of a Multi-Omics-Based Anti-Inflammatory Diet

Limitations in the implementation of a multi-omics-based anti-inflammatory diet primarily concern patients with obesity and/or metabolic syndrome (MetS) who present with gastrointestinal symptoms that restrict tolerance to high-residue versions of anti-inflammatory dietary patterns, particularly in individuals with functional bowel disorders (mainly IBS) and in a subset of patients with inflammatory bowel disease (IBD). In IBS, a key limiting mechanism is excessive fermentation and the osmotic effect of short-chain carbohydrates (FODMAPs), which may exacerbate abdominal pain, bloating, and altered bowel habits. Such symptoms are typically triggered by the consumption of legumes, coarse whole grains, allium vegetables, and selected fruits [172]. For this reason, in patients with active IBS symptoms, a multi-omics-based anti-inflammatory diet should be introduced in a modified form that includes temporary FODMAP restriction, ideally as a staged intervention followed by gradual reintroduction and personalization, and with a preference for soluble fibre rather than abrupt increases in total fibre intake or prebiotic supplementation [172,173].

In IBD, an additional and significant limitation is the complexity and inter-individual variability of dysbiosis, along with the recognition that assessment of “microbiome health” should not rely solely on taxonomic composition. Zielińska et al. [174] emphasize the importance of evaluating the functional metabolic potential of the microbiota rather than taxonomic profiles alone, supporting the notion that universal increases in fermentable fibre or aggressive prebiotic interventions may not provide predictable benefits for all patients and therefore require individualized planning and close clinical monitoring. In clinical practice, particularly during disease exacerbations, nutritional tolerance and safety remain primary priorities. Strategies based on microbiota modulation should be applied cautiously to avoid worsening gastrointestinal symptoms or compromising cardiometabolic treatment goals relevant to obesity and MetS.

## 8. Conclusions

An anti-inflammatory dietary pattern constitutes a key component of non-pharmacological management in obesity and metabolic syndrome (MetS), as it targets fundamental pathophysiological mechanisms underlying these conditions, including chronic low-grade inflammation, adipose tissue dysfunction, insulin resistance, and disturbances of the gut–metabolic axis. The integration of omics data, encompassing gut microbiota composition and function (metagenomics), adipokine profiles, nutrigenomics, epigenetics, as well as related transcriptomic and metabolomic layers, enables more precise characterization of a patient’s metabolic phenotype and may support the development of precision nutrition strategies aimed at preventing chronic disease and optimizing individual responses to dietary interventions.

A common denominator across practical anti-inflammatory dietary models is an emphasis on dietary quality rather than solely on macronutrient quantity, with particular attention to lipid profile. In clinical practice, reducing total dietary fat intake (to below approximately 30% of total energy) is recommended primarily through decreasing saturated fatty acids, trans fats, and omega-6 fatty acids, while increasing the intake of long-chain omega-3 PUFA (EPA/DHA) and plant-based sources of α-linolenic acid (ALA). Accordingly, anti-inflammatory diets for patients with obesity and MetS should include greater proportions of lean protein sources and low-glycemic index carbohydrates rich in dietary fibre to enhance satiety. The use of low-glycemic index and low-glycemic load carbohydrates, characterized by high fibre content, particularly fermentable fractions, modulates the composition and metabolic activity of the gut microbiota. Another important component is maintaining a high intake of bioactive dietary constituents with antioxidant and immunomodulatory potential, especially polyphenols. Introducing these qualitative dietary changes in an ad libitum context, through enhanced satiety signalling, may promote a negative energy balance and subsequent weight reduction.

**Figure 1:**
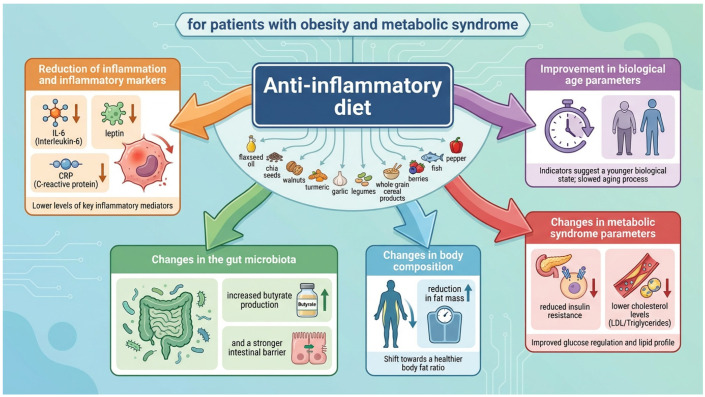
Multi-Omics and Clinical Impact of an Anti-Inflammatory Diet in Patients with Obesity and Metabolic Syndrome. The figure illustrates the potential effects of an anti-inflammatory diet on inflammatory status, gut microbiota, body composition, metabolic syndrome parameters, and biological age. Small upward arrows indicate an increase, whereas small downward arrows indicate a decrease in the indicated parameter. The large colored arrows represent the direction of the potential influence of the anti-inflammatory diet on each domain. The figure was generated using Gemini 3 Pro Image (Nano Banana Pro; Google LLC, Mountain View, CA, USA), based on the conclusions prepared by the author of this manuscript.

To improve feasibility and facilitate long-term adherence, dietary recommendations should be flexible and based on practical food substitutions. Within an anti-inflammatory framework, limiting red and processed meats is advised in favor of lean poultry, low-fat dairy products, and marine fish as primary sources of EPA/DHA. Plant-derived fats should be used predominantly in cold applications, with preference given to oils characterized by favourable fatty acid profiles (e.g., flaxseed, chia seed, and camelina oils), while limiting oils high in saturated fatty acids (e.g., coconut oil) and omega-6 fatty acids (e.g., sunflower and peanut oils). The diet should incorporate nuts and seeds, particularly flaxseed, chia seeds, and walnuts as sources of ALA, fibre, and phenolic compounds, serving as healthier alternatives to salted snack foods. The primary sources of dietary energy should include whole-grain cereal products such as coarse groats, brown rice, whole-grain durum semolina pasta, oats, whole-grain bread, and low-glycemic legumes, which provide fibre, resistant starch, and plant protein while reducing reliance on highly processed foods. To enhance sensory acceptance and reduce sodium intake, herbs, spices, and flavouring components (e.g., turmeric combined with black pepper, garlic, vinegar, and chili peppers) should be used as substitutes for excessive salt. The diet should be rich in a wide variety of vegetables, including fermented vegetable products such as unpasteurized sauerkraut, as well as fruits, particularly berries such as raspberries and strawberries, which serve as sources of fibre and polyphenols and as practical alternatives to high-sugar foods. Fruits should preferably be consumed whole rather than in the form of juices, in order to preserve fibre content and support a lower glycaemic response. The dietary recommendations presented in this review, along with their proposed multi-omics and clinical impact in patients with obesity and metabolic syndrome, are summarized in Figure 1.

Such a dietary model has strong clinical justification in the management of obesity and MetS, as it can simultaneously modulate multiple pathophysiological axes, including gut barrier function and microbiota-derived metabolites, lipid metabolism and inflammatory status, as well as gene expression regulation through epigenetic pathways. These combined effects may contribute to improved metabolic outcomes and a reduction in cardiovascular risk. Nevertheless, well-designed clinical intervention studies employing a multi-omics-based anti-inflammatory dietary framework are required to confirm its impact across specific domains of human health. At present, the most consistent human evidence concerns dietary effects on systemic inflammation, insulin sensitivity, and selected adipokines, whereas causal links involving microbiota-derived metabolites, DNA methylation, and full multi-omics integration remain promising but less firmly established. 

## Data Availability

No new data were created or analyzed in this study. Data sharing is not applicable to this article.
